# Perioperative Mass Transfusion Affects In-Hospital but Not Follow-Up Survival in Patients with Acute Type A Aortic Dissection

**DOI:** 10.3390/medicina59101825

**Published:** 2023-10-13

**Authors:** Julia Benk, Tim Berger, Roman Gottardi, Tim Walter, Stoyan Kondov, Bartosz Rylski, Martin Czerny, Maximilian Kreibich

**Affiliations:** Department of Cardiovascular Surgery, Heart Centre Freiburg University, Faculty of Medicine, University of Freiburg, 79106 Freiburg, Germany; julia.benk@uniklinik-freiburg.de (J.B.); tim.berger@uniklinik-freiburg.de (T.B.); roman.gottardi@uniklinik-freiburg.de (R.G.); tim.walter@uniklinik-freiburg.de (T.W.); stoyan.kondov@uniklinik-freiburg.de (S.K.); bartosz.rylski@uniklinik-freiburg.de (B.R.); martin.czerny@uniklinik-freiburg.de (M.C.)

**Keywords:** type A aortic dissection, mass transfusion, postoperative outcome

## Abstract

*Background and Objectives*: The aim of this study was to analyze the influence of mass transfusion on the postoperative outcome and survival in patients presenting with acute Type A aortic dissection. *Materials and Methods*: Between 2002 and 2020, a total of 505 patients were surgically treated for an acute Type A aortic dissection. Mass transfusion was defined as the peri- and postoperative replacement by transfusion of 10 units. Patient characteristics and outcomes were analyzed and compared between patients with and without mass transfusion. *Results*: Mass transfusion occurred in 105 patients (20%). The incidences of symptomatic coronary malperfusion (*p* = 0.017) and tamponade (*p* = 0.043) were higher in patients with mass transfusion. There was no statistically significant difference in the distal extension of the aortic dissection between the two groups. A valved conduit was significantly more common in patients with mass transfusion (*p* = 0.007), while the distal aortic repair was similar between the two groups. Cardiopulmonary bypass time (*p* < 0.001), cross clamp time (*p* < 0.001) and in-hospital mortality were significantly higher in patients with mass transfusion (*p* < 0.001), but the survival after discharge (landmark-analysis) showed equal survival between patients with and without mass transfusion (log rank: *p* = 0.4). Mass transfusion was predictive of in-hospital mortality (OR: 3.308, *p* < 0.001) but not for survival after discharge (OR: 1.205, *p* = 0.661). *Conclusions*: Mass transfusion is necessary in many patients with acute Type A aortic dissection. These patients present sicker and require longer surgery. However, mass transfusion does not influence survival after discharge.

## 1. Introduction

Blood transfusions are common in cardiac surgery but are associated with reduced in-hospital and long-term survival [1]. In fact, about 20% of the transfused packed red blood cells in the United States are used in cardiac surgery [2]. In patients with acute Type A aortic dissection, blood transfusions are an almost inevitable part of perioperative management. The dysregulation of the coagulation system due to the aortic dissection itself and the invasive nature of aortic surgery using cardiopulmonary bypass and deep hypothermia for brain protection are the two key reasons for the high need for blood transfusion in these patients [3]. Although blood transfusions are potentially life-saving in the emergency setting, there are several risks, like transfusion-related acute lung injury, transfusion reactions, infectious complications and immunomodulation, potentially leading to a higher rate of cancer [1,2]. Therefore, the aim of this study was to investigate the influence of mass transfusion on the in-hospital outcomes and follow-up survival of patients undergoing immediate surgery for acute Type A aortic dissection.

## 2. Materials and Methods

### 2.1. Ethics Statement

Our institutional review committee (University Hospital Freiburg) approved this retrospective study (IRB number: 20-1302), and the need for informed consent was waived because of the retrospective nature of the study.

### 2.2. Patients and Follow-Up Protocol

Our study population consists of 505 patients who were surgically treated for an acute Type A aortic dissection between 2002 and 2020 in one aortic center. All patients were followed up for a total of 108 patient-years, with a median follow-up of 13 [3, 32] months. Over the last few years, we have implemented a dedicated aortic clinic for routine follow-up examinations of all aortic patients. Routinely, patients are followed up after six months, twelve months and yearly thereafter in our aortic clinic.

### 2.3. Definition of Groups and Parameters

Data were collected retrospectively using our center’s prospectively maintained aortic databases. Baseline and aortic characteristics, previous aortic procedures, intraoperative details, clinical outcomes and follow-up data were evaluated. Mass transfusion was defined as the perioperative transfusion of 10 or more packed red blood cells. We compared clinical features and outcomes after operation between patients with and without mass transfusion. The modified Rankin Scale (mRS) was used to classify the severity of a postoperative stroke [4]. All strokes were assessed by consulting neurologists. Cardiogenic shock was defined as persistent hypotension (systolic blood pressure < 80 mm Hg) with severe reduction in cardiac index. The type, entry and malperfusion (TEM) classification system [5] was used to describe the dimension of the aortic dissection. In this study, only patients with Type A aortic dissection were included. The categories of the primary entry are characterized as follows: E0 = no visible entry; E1 = entry in the ascending aorta; E2 = entry in the aortic arch; E3 = entry in the descending aorta. The categories of the end-organ malperfusion are characterized as follows: M0 = no radiological or clinical signs of malperfusion; M1 = dissection of at least one main coronary artery with (M1+) or without (M1−) indicators of cardiac ischemia; M2 = dissection of at least one supra-aortic vessel or aortic arch true lumen collapse with (M2+) or without (M2−) clinical symptoms of cerebral or upper extremity; M3 = dissection or false lumen origin of at least one visceral, renal or one iliac artery or aortic true lumen collapse entailing functional closure of at least one visceral, renal or iliac artery offspring, with (M3+) or without (M3−) clinical symptoms of bowel, kidney or lower-extremity ischemia.

### 2.4. Surgical Approach

For all patients, a previously described standardized integrated surgical management was applied [6]. Aortic repair always included replacing the entire ascending aorta, with or without root replacement (valve conduit or valve sparring root replacement). The distal repair included isolated ascending, hemiarch, or complete arch replacement, depending on the entry tear location and aortic diameter. During the later study period, the frozen elephant trunk technique became our routine method for total aortic arch replacement. In this case, anastomosis was routinely performed in zone 2, and we aimed for no oversizing of the stent-graft in relation to the true lumen diameter of the dissected downstream aorta. Aortic arch operations were performed in hypothermic circulatory arrest with selective antegrade cerebral perfusion and open distal anastomosis.

### 2.5. Blood Management

In our institution, the usual intraoperative transfusion trigger for packed red blood cells is a hemoglobin < 8 g/dL. However, during the long study period of 18 years the transfusion strategy may have changed from a more liberal to an actual more restrictive regimen nowadays. Today, we follow the recommendations of the Task Force on Patient Blood Management for Adult Cardiac Surgery of the European Association for Cardio-Thoracic Surgery (EACTS) and the European Association of Cardiothoracic Anaesthesiology (EACTA), which suggests transfusing packed red blood cells on the basis of the clinical condition of the patient rather than on a fixed hemoglobin threshold [7]. Usually, the trigger for transfusion of platelet concentrates in our institution is a platelet count below 50 (10^9^/L) in patients with symptoms of acute bleeding, as recommended in the current guidelines [7]. The common trigger for transfusion of fresh-frozen plasma was to reverse the effect of vitamin K antagonists before the emergency operation or the intraoperative transfusion of more than six packed red blood cells. During operation, the coagulation was usually monitored via standard laboratory results like partial thromboplastin time (PTT), international normalized ratio (INR), fibrinogen level, platelet count and viscoelastic point of care tests.

### 2.6. Statistical Analysis

Data are presented as absolute and relative frequency or as median [first quartile, third quartile]. The Student’s T-test or the Mann–Whitney U-test were used to compare continuous variables as appropriate. Categorical variables were compared using the Chi-squared test with the calculation of exact values. In case of small group sizes (*n* < 5), Fisher’s exact test was used. The influence of the number of transfused packed red blood cells on the probability of in-hospital mortality was assessed with logistic regression analysis. Multivariable logistic regression was performed to analyze the influence of selected variables (sex, age, mass transfusion, shock on admission and malperfusion) on in-hospital mortality. A Cox regression model with clinically selected variables (sex, age, mass transfusion, shock on admission and malperfusion) was used to assess the influence of selected variables on the survival after discharge. Overall survival and survival after discharge (landmark point) were analyzed using the Kaplan–Meier method.

## 3. Results

### 3.1. Patient Characteristics

All patient characteristics are summarized in Table 1. From 505 patients treated for acute Type A aortic dissection, 403 patients (80%) did not receive mass transfusion and 102 patients (20%) received ten or more packed red blood cells during or after operation. Mean age was 65 years and 303 patients (60%) were male. The incidence of diabetes mellitus type 2 or connective tissue disease was significantly increased in patients requiring mass transfusion in this study.

### 3.2. TEM Classification and Clinical Presentation

Most of the entries (69%) were located in the ascending aorta. There was no statistically significant difference in entry location between patients with or without mass transfusion. Using the TEM classification, significantly more patients with coronary malperfusion required mass transfusion in this study. There was no statistically significant difference in the other types of malperfusion between patients with or without mass transfusion. On admission, 118 patients (23%) presented with cardiogenic shock and 147 patients (29%) showed a clinical sign of malperfusion. Patients with aortic valve regurgitation on admission were less likely to receive mass transfusion during operation. All details are illustrated in Table 2.

### 3.3. Dissection Extent

There was no statistically significant difference with regard to the distal extent of the aortic dissection between the two groups. All details are summarized in Table 3.

### 3.4. Surgical Details

Cannulation for cardiopulmonary bypass was performed via subclavian artery in 399 patients (79%). Patients with cannulation of the femoral artery were more likely to receive mass transfusion during operation. Concerning the proximal repair, patients with a Bentall procedure received significantly more mass transfusions. Relating to the distal aortic repair, there was no statistically significant difference between the two groups. Patients with longer cardiopulmonary bypass time, longer cross clamp time, longer hypothermic circulatory arrest and lower body temperature during operation did receive significantly more mass transfusions. All surgical details are summarized in Table 4.

### 3.5. Outcome Characteristics, Complications and Transfused Blood Products

**Figure 1:**
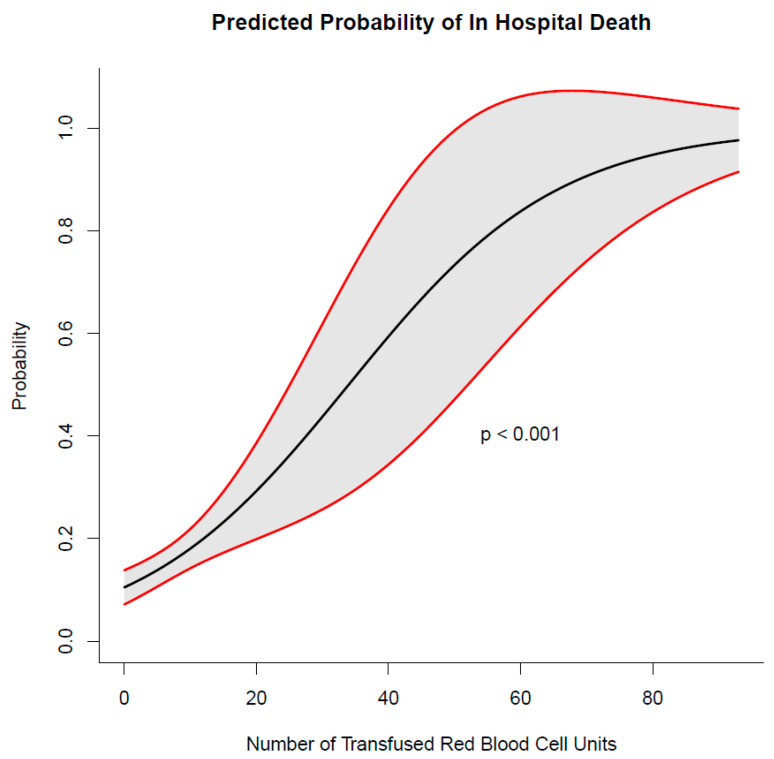
Increasing probability for in-hospital mortality with every transfused packed red blood cell product.

Re-operation because of bleeding was the most common complication in 100 patients (20%) followed by postoperative stroke in 81 patients (16%). Patients receiving intraoperative mass transfusion had significantly higher incidences of postoperative dialysis or tracheostomy. All outcome characteristics, complications and transfused blood products are summarized in Table 5. The probability for in-hospital mortality increased significantly with every transfused packed red blood cell product (Figure 1).

### 3.6. Logistic Regression Model

Our logistic regression model showed that age, mass transfusion, shock on admission and any organ malperfusion were predictive for in-hospital mortality. The full model is summarized in Table 6.

### 3.7. Long-Term Survival and Landmark Analysis

**Figure 2:**
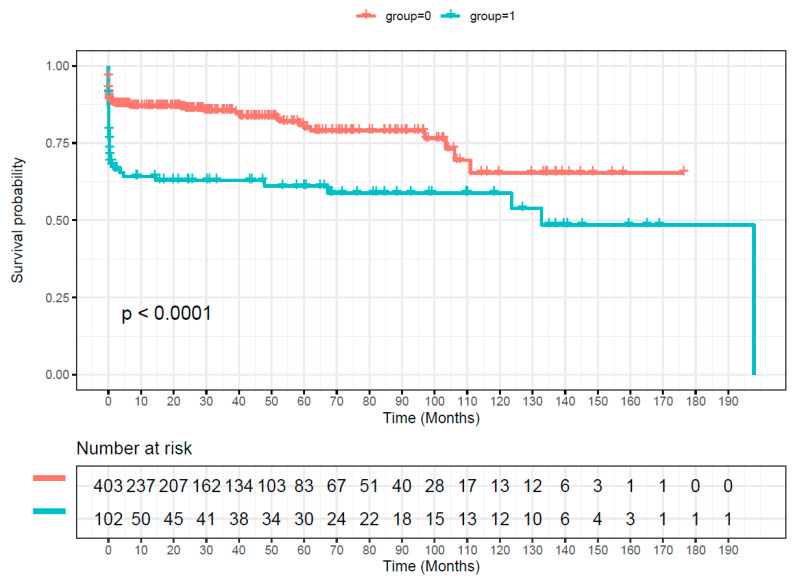
Kaplan–Meier curve showing our cohort’s overall survival. Group 0 (red) = no mass transfusion. Group 1 (green) = mass transfusion.

**Figure 3:**
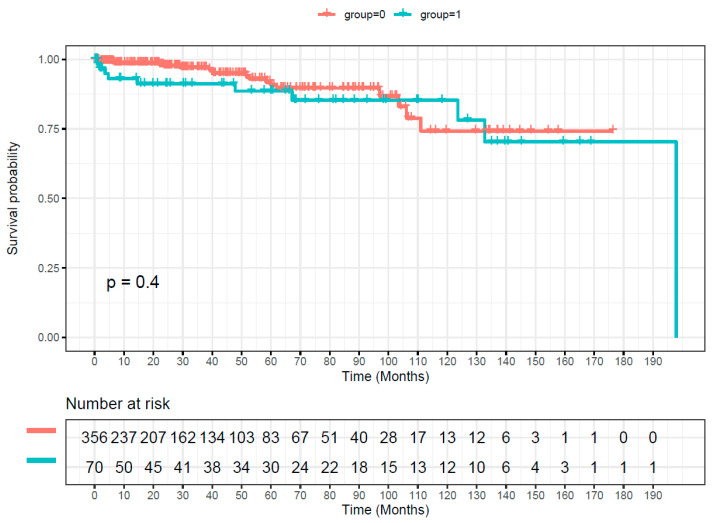
Kaplan–Meier curve showing our cohort’s overall survival after discharge (landmark point). Group 0 (red) = no mass transfusion. Group 1 (green) = mass transfusion.

Overall survival was significantly worse in patients with mass transfusion (Figure 2, log rank: *p* < 0.001). However, the landmark analysis showed that survival after discharge (landmark point) did not differ between the two groups (Figure 3, log rank: *p* = 0.4). In addition, our Cox model of discharged patients (landmark point) showed that mass transfusion was not a statistically significant predictor for late mortality.

## 4. Discussion

This study’s most important findings are that (i) mass transfusion is very common in patients with acute Type A aortic dissection and (ii) mass transfusion is more common in patients with malperfusion and shock and in cases of more extensive surgery, but (iii) mass transfusion does not influence late survival after discharge.

Our study population is comparable to other investigations [3,8,9] with 60% male patients, average age of 65 [54, 74] years and common cardiovascular risk factors. From 505 patients treated for acute Type A aortic dissection, 102 patients (20%) received ten or more packed red blood cells during or after operation. Another large study with more than 3000 patients even reported mass transfusion rates of 37% during surgical repair of acute type A aortic dissection [9]. Patients with diabetes mellitus type 2 (*n* = 34) or connective tissue disease (*n* = 4) had a significant increased risk of mass transfusion in this study. It seems plausible that patients with a connective tissue disease more commonly received aortic root replacement, a variable that was also more common in patients with mass transfusion, because of the worse long-term outcome in these patients, if root replacement is not performed during the repair of Type A dissection [10]. The role of diabetes mellitus remains unclear and warrants further research in this field.

On admission, 118 patients (23%) presented with cardiogenic shock and 109 patients (22%) showed a clinical relevant cardiac tamponade. The latter was predictive for mass transfusion (*p* = 0.043), as well as coronary malperfusion, with (M1+) or without (M1−) indicators of cardiac ischemia (M1 minus *p* = 0.001, M1 plus *p* = 0.017). This was also observed in other studies [9,11,12], and can be explained by the more complex surgery with aortocoronary bypass grafting. Tamponade on admission does not only affect mass transfusion, but worsens the outcome of the patients with higher mortality [9,11,13]. Interestingly patients with aortic valve regurgitation on admission were less likely to receive mass transfusion during operation, maybe because not all of these patients needed aortic valve replacement or valved conduit, which would have extended the operation. Frequently, the valve itself is not disturbed, so aortic valve resuspension was performed without significant enlarging the surgery.

Most of the entries of the dissections were located in the ascending aorta *n* = 347 (69%), followed by the aortic arch *n* = 71 (14%), which is comparable to other studies [5]. The dissection extended to the abdominal aorta in 256 (51%) patients. There was no statistically significant difference with regard to the distal extent of the aortic dissection or the location of the entry between patients with or without mass transfusion. Hence, this study shows that a tear-oriented approach with replacement of the aortic arch in a standardized method does not influence the incidence of mass transfusion. Although total arch replacement enlarges the operation, Shrestha et al. could show that the frozen elephant trunk procedure is a safe treatment option in patients with Type A dissection and entry tear in the aortic arch [14].

Patients with cannulation via the femoral artery were more likely to receive mass transfusion compared to our standard cannulation via the subclavian artery. These patients usually present in a critical state with instable hemodynamics, and require rapid stabilization with cardiopulmonary bypass [15]. Concerning the proximal repair, mass transfusion was more prevalent in patients requiring aortic root replacement by Bentall procedure. This may be explained by the longer and more complex surgery, compared to a simple supracoronary graft, but also by the higher prevalence of coronary malperfusion in this group. Valve-sparing aortic root repair was only performed in four patients (4%) with mass transfusion, compared to 39 patients (10%) in the group without mass transfusion. This may indicate that the surgeons performing valve-sparing aortic root repair were more experienced and therefore caused fewer complications, as we could show in previous studies [16]. Patients with mass transfusions had significantly longer cardiopulmonary bypass time, cross clamp time and hypothermic circulatory arrest, as well as lower body temperature, during operation. This is in line with other studies [3,9,12] and confirms that the need for extended periods of heart lung machine and hypothermia aggravates the coagulation system, which is already restricted by the aortic dissection itself [3,12].

During the postoperative stay, patients with mass transfusion were significantly more likely to develop complications such as acute renal failure with dialysis or long-term ventilation and tracheostomy with a consequently higher risk of dying during the hospital stay. These findings were reported in prior studies and are most likely explained by the potentially toxic effects of blood transfusions like inflammation, constricted oxygen delivery and hemostasis dysfunction [8,9,11,12]. After all, mass transfusion in this study may be seen as a surrogate marker for more complex aortic dissections, requiring more extensive surgery with a higher risk of adverse postoperative events.

In our study, overall survival was significantly worse in patients with mass transfusion (*p* < 0.001), as described earlier by other authors [1,9,12]. The probability for in-hospital mortality in this cohort increased significantly with every transfused packed red blood cell product. Nevertheless, this has to be interpreted carefully, because the patients, which need blood transfusions, are most frequently already in a poor state, compared to patients, which do not need blood transfusions [1]. In fact, our logistic regression model showed that mass transfusion, age, shock on admission and any organ malperfusion were predictive for in-hospital mortality. This indicates that the influence of mass transfusion on in-hospital mortality was probably also influenced by the clinical presentation of the patients.

Interestingly, in this study, the survival after discharge did not differ between patients with or without mass transfusion (*p* = 0.4). Many studies report a correlation between mass transfusion and worst long-time survival [1,9,11,12], but there are others investigating cardiovascular patients undergoing surgical hip fracture repair that indicate that liberal blood transfusion did not affect mortality [17]. Another study examining the outcome after coronary artery bypass surgery observed no difference in the long-term outcomes between patients with and without transfusions over a period of eight years, but they excluded patients with mass transfusions [18]. Definitely, further studies are necessary to clarify this issue. After all, the bleeding patient with a Type A dissection requires transfusions to survive, and the data here suggest no negative effect on the long-term survival once the patient has survived the acute bleeding event.

### Limitations and Strengths

Our study is limited by its retrospective nature, leading to confounding, risk of type II error and high heterogeneity of the cohort. However, this investigation contributes valuable knowledge on the effects of mass transfusion during surgical repair for acute Type A dissection on the outcome of patients.

## 5. Conclusions

This study highlights the common need for mass transfusion in patients undergoing surgical repair of an acute Type A aortic dissection. Transfusions are more common in patients with malperfusion and shock, as well as in cases of more extensive surgery, and significantly impact in-hospital mortality. However, mass transfusion does not influence late survival after discharge.

## Figures and Tables

**Table 1:** Patient characteristics.

	All Patients (*n* = 505)	No Mass Transfusion (*n* = 403)	Mass Transfusion (*n* = 102)	*p*
Age (years)	65 [54, 74]	65 [53, 74]	65 [58, 73]	0.463
Male	303 (60)	244 (61)	59 (58)	0.652
Diabetes mellitus type 2	34 (7)	21 (5)	13 (13)	0.010
Hyperlipidemia	185 (37)	156 (39)	29 (28)	0.051
Hypertension	403 (80)	325 (81)	78 (76)	0.471
History of stroke	45 (9)	32 (8)	13 (13)	0.170
History of renal failure	55 (11)	42 (10)	13 (13)	0.593
Dialysis	13 (3)	9 (2)	4 (4)	0.308
COPD	34 (7)	25 (6)	9 (9)	0.375
Coronary artery disease	82 (16)	61 (15)	21 (21)	0.175
Bicuspid aortic valve	26 (5)	19 (5)	7 (7)	0.449
Connective tissue disease	4 (1)	1 (0.3)	3 (3)	0.027

Values are *n* (%) or median [first quartile, third quartile]. COPD, chronic obstructive pulmonary disease.

**Table 2:** TEM classification and clinical presentation.

	All Patients (*n* = 505)	No Mass Transfusion (*n* = 403)	Mass Transfusion (*n* = 102)	*p*
E0	73 (14)	59 (15)	14 (14)	0.876
E1	347 (69)	280 (69)	67 (66)	0.475
E2	71 (14)	55 (14)	16 (16)	0.633
E3	14 (3)	9 (2)	5 (5)	0.172
M0	227 (45)	190 (47)	37 (36)	0.058
M1 minus	16 (3)	7 (2)	9 (9)	0.001
M1 plus	59 (12)	40 (10)	19 (19)	0.017
M2 minus	104 (21)	83 (21)	21 (21)	1.000
M2 plus	88 (17)	67 (17)	21 (21)	0.379
M3 minus	68 (13)	53 (13)	15 (15)	0.745
M3 plus	73 (14)	56 (14)	17 (17)	0.528
**Clinical presentation**				
Shock	118 (23)	89 (22)	29 (28)	0.191
Tamponade	109 (22)	79 (20)	30 (29)	0.043
Aortic valve regurgitation	103 (20)	84 (21)	13 (13)	0.024

Values are *n* (%). TEM: type, entry site, malperfusion.

**Table 3:** Dissection extent.

	All Patients (*n* = 505)	No Mass Transfusion (*n* = 403)	Mass Transfusion (*n* = 102)	*p*
Brachiocephalic trunk	255 (51)	196 (49)	59 (58)	0.055
Right subclavian artery	62 (12)	45 (11)	17 (17)	0.123
Right common carotid artery	135 (27)	100 (25)	35 (34)	0.061
Left common carotid artery	132 (26)	103 (26)	29 (28)	0.612
Left subclavian artery	115 (23)	90 (22)	25 (25)	0.594
Descending aorta	304 (60)	240 (60)	64 (63)	0.348
Abdominal aorta	256 (51)	198 (49)	58 (57)	0.088

Values are *n* (%).

**Table 4:** Surgical details.

	All Patients (*n* = 505)	No Mass Transfusion (*n* = 403)	Mass Transfusion (*n* = 102)	*p*
**Cannulation**				
Subclavian artery	399 (79)	325 (81)	74 (73)	0.167
Aorta	34 (7)	30 (7)	4 (4)	0.270
Femoral artery	73 (14)	49 (12)	24 (24)	0.005
**Proximal repair**				
Aortic valve resuspension	346 (69)	279 (69)	67 (66)	0.475
Bentall procedure	83 (16)	57 (14)	26 (25)	0.007
Valve-sparing aortic root repair	43 (9)	39 (10)	4 (4)	0.073
Wheat procedure	25 (5)	20 (5)	5 (5)	1.000
**Distal repair**				
Ascending aorta	209 (41)	166 (41)	43 (42)	0.911
Hemiarch	220 (44)	180 (45)	40 (39)	0.317
Total arch	47 (9)	33 (8)	14 (14)	0.125
Cardiopulmonary bypass time in minutes	182 [144, 226]	172 [138, 214]	213 [165, 272]	<0.001
Cross clamp time in minutes	98 [75, 132]	95 [73, 122]	121 [87, 161]	<0.001
Hypothermic circulatory arrest in minutes	17 [0, 33]	13 [0, 33]	23 [0, 33]	0.036
Lowest body temperature in °Celsius	25 [23, 27]	25 [24, 28]	24 [22, 25]	<0.001

Values are *n* (%) or median [first quartile, third quartile].

**Table 5:** Outcome characteristics, complications and transfused blood products.

	All Patients (*n* = 505)	No Mass Transfusion (*n* = 403)	Mass Transfusion (*n* = 102)	*p*
**Complications**				
Re-operation because of bleeding	100 (20)	52 (13)	48 (47)	<0.001
Dialysis	54 (11)	32 (8)	22 (22)	<0.001
Tracheostomy	49 (10)	31 (8)	18 (18)	0.004
Percutaneous endoscopic gastrostomy	16 (3)	13 (3)	3 (3)	1.000
Stroke	81 (16)	60 (15)	21 (21)	0.175
In-hospital death	79 (16)	47 (12)	32 (31)	<0.001
**Blood products**				
RBCs	5 [3, 8]	4 [2, 6]	14 [12, 20]	<0.001
FFPs	6 [4, 10]	6 [4, 8]	16 [10, 22]	<0.001
PCs	3 [2, 4]	2 [2, 4]	4 [2, 7]	<0.001

Values are *n* (%) or median [first quartile, third quartile]. RBC: packed red blood cells; FFP: fresh frozen plasma; PC: platelet concentrate.

**Table 6:** Logistic regression model.

Logistic Regression Analysis: In-Hospital Death			
Variable	*p*	OR	95% CI
Sex	0.428	0.806	0.473–1.374
Age	0.024	1.024	1.003–1.046
Mass transfusion	<0.001	3.308	1.892–5.785
Shock	<0.001	3.889	2.237–6.763
Malperfusion	0.010	2.077	1.195–3.610

OR, odds ratio; CI, confidence interval.

## Data Availability

The datasets discussed in this article are not readily available because of our institutional review board’s requirements. Reasonable individual requests will be evaluated by the corresponding author.
